# IL-33/ST2 Axis Protects Against Traumatic Brain Injury Through Enhancing the Function of Regulatory T Cells

**DOI:** 10.3389/fimmu.2022.860772

**Published:** 2022-03-30

**Authors:** Di Xie, Wanying Miao, Fei Xu, Chunling Yuan, Sicheng Li, Chujun Wang, Aditi Junagade, Xiaoming Hu

**Affiliations:** ^1^Department of Neurology, School of Medicine, University of Pittsburgh, Pittsburgh, PA, United States; ^2^Geriatric Research, Education and Clinical Center, Veterans Affairs Pittsburgh Health Care System, Pittsburgh, PA, United States

**Keywords:** IL-33, ST2, regulatory T cells, traumatic brain injury, neuroprotection

## Abstract

Traumatic brain injury (TBI) is a devastating condition due to its long-term sequelae on neurological functions. Inflammatory responses after TBI are critical for injury expansion and repair. Recent research in central nervous system (CNS) disorders reveals the importance of IL-33 and its receptor (ST2) as an alarmin system to initiate immune responses. This study explored the role of IL-33/ST2 signaling in TBI. TBI was induced in adult male C57BL/6J mice using a controlled cortical impact (CCI) model. We found that the expression of IL-33 increased in the injured brain and blood, and ST2 was elevated in the circulating and infiltrating regulatory T cells (Tregs) early after TBI. ST2 deficient mice exhibited reduced Treg numbers in the blood and brain 5 days after TBI. The brain lesion size was enlarged in ST2 knockout mice, which was accompanied by deteriorated sensorimotor function 5 days after TBI. In contrast, post-TBI treatment with IL-33 (2 μg/30 g body weight, intranasal) for 3 days significantly reduced brain lesion size and improved neurological functions 5 days after TBI. Meanwhile, IL-33 treatment increased ST2 expression in circulating and brain infiltrating Tregs. To further explore the involvement of Tregs in IL-33/ST2-mediated neuroprotection, Tregs were depleted by CD25 antibody injection. The absence of Tregs significantly reduced the protective effect of IL-33 after TBI. *In vitro* study confirmed that IL-33 (50 ng/ml) increased the production of IL-10 and TGFβ from activated Tregs and boosted the inhibitory effect of Tregs on T effector cell proliferation. Taken together, this study suggests that the activation of IL-33/ST2 signaling reduces brain lesion size and alleviates functional deficits after TBI at least partially through regulating the Treg response. IL-33 may represent a new immune therapeutic strategy to improve TBI outcomes.

## Introduction

Traumatic brain injury (TBI) is a devastating condition due to its high mortality and morbidity. It has hitherto not been possible to alleviate the long-term neurological deficits after TBI. Data from the Centers for Disease Control report around 1.7 million TBI patients annually (1). An estimated 5.3 million people are living with permanent TBI-related motor and cognitive disabilities or mood disorders in the United States (1). In addition, the incidences of neurodegenerative diseases, including Alzheimer’s Disease and Parkinson’s Disease increase among TBI patients (2).

TBI is a progressive CNS injury. Pathological changes after TBI include the acute immediate brain lesion and secondary damages occur days or weeks after initial injury (3). Post-TBI inflammation is a known process that contributes to the progress of injury expansion and repair. Large amounts of immune cells, including resident microglia, astrocytes, infiltrating macrophages, neutrophils and lymphocytes, are activated and recruited to the site of injury. The activation of these cells, although essential for debris clearance and brain repair, has to be well-regulated to avoid unwanted side effects from the upsurge of inflammatory factors. Recent breakthroughs in understanding immune regulation during CNS pathology highlight the contributions of regulatory T cells (Tregs) to brain injury and repair through immunomodulation. Further understanding the mechanisms of post-TBI immune responses may identify rational therapeutic targets with a large window of opportunity.

IL-33 belongs to the IL-1 superfamily and resides in the nucleus of normal cells. Residential CNS cells, such as astrocytes and neurons, release IL-33 to regulate microglial function during brain development and in a mature normal brain (4, 5). Damages to cells trigger the release of IL-33 from the nucleus, which activates immune responses. Research in CNS disorders advocates the importance of IL-33 as an alarmin protein to initiate immune response after brain lesions. Our previous study shows that IL-33 is released from oligodendrocytes and astrocytes early after stroke, and the activation of IL-33 receptor, ST2, protects against acute ischemic neuronal and oligodendrocyte loss (6, 7). The activation of IL-33/ST2 signaling also adjusts the adaptive immune responses after brain injuries. For example, IL-33 treatment induces Th2-type responses and reduces post-stroke inflammation (8). IL-33/ST2 signaling also expands the number of Tregs and enhances their beneficial effects in the ischemic brain (9–11). Clinical studies document that serum concentrations of IL-33 and soluble ST2 significantly increase in TBI patients *vs.* controls and correlate with worse prognosis after TBI (12, 13). The exact role of IL-33/ST2 signaling in TBI, however, is not thoroughly studied.

In this study, we found that IL-33 expression increased in brain and blood, and expression of ST2 was elevated in circulating Tregs and brain infiltrating Tregs early after TBI. The activation of IL-33/ST2 signaling reduced brain lesion size and improved functional performance after TBI at least partially through regulating Treg activity. IL-33 may represent a new immune therapeutic strategy to improve TBI outcomes.

## Methods

### Animals

C57BL/6J (wild-type, WT) mice were purchased from the Jackson Laboratory (Bar Harbor, Maine). ST2 KO mice, a gift from Dr. A. McKenzie (Medical Research Council as part of UKRI, U.K), were bred for experimental use at the University of Pittsburgh. Mice were maintained under a 12h – 12h light-dark cycle with food and water provided *at libitum*. All animal procedures were approved by the University of Pittsburgh Institutional Animal Care and Use Committee and performed following the *Guide for the Care and Use of Laboratory Animals*. For Treg depletion, 300 μg anti-CD25 antibody (Thermo Fisher, Pittsburgh, PA) was diluted in PBS and injected ip 2 days prior to CCI. Control mice received the same amount of IgG2a isotype antibody.

### Murine Models of Traumatic Brain Injury

TBI was induced by unilateral controlled cortical impact (CCI) as previously described (14). Briefly, mice were randomly assigned to sham and TBI groups with different treatments. All animals were anesthetized with 1.5% isoflurane in a 30% O_2_/70% N_2_O mixture under spontaneous breathing. Mice were drilled with a right parietal craniotomy (diameter of 3.5 mm; centered 0.5 mm anterior and 2.0 mm lateral to Bregma). The CCI was produced with a pneumatically driven CCI device (Precision Systems and Instrumentation) using a 3 mm flat-tipped impounder (velocity, 3.75 m/s; duration, 150 ms; depth, 1.5 mm). After CCI, the skin incision was sealed. Rectal temperature was maintained at 37°C ± 0.5°C during surgery and for up to 30 minutes after CCI using a heating pad. Sham animals were subjected to all aspects of the protocol (surgery, anesthesia, craniotomy, recovery) except for CCI. CCI animal models and all outcome assessments were performed by investigators who were blinded to the mouse genotype and the group assignments.

### Intranasal Administration of IL-33

Animals were randomly assigned to intranasally receive recombinant IL-33 (Enzo, 2μg/30g body weight), or vehicle control treatment at 2h, 24h, and 48h after CCI. Mice were anesthetized as described above and placed in a supine position. The solution was applied into each nostril 3 times (~5 μl into each nostril for each infusion) with an interval of 5 minutes.

### Behavioral Test

#### Adhesive Removal Test 

Adhesive removal test was performed to assess tactile responses and sensorimotor function. Adhesive tapes (2 mm × 3 mm) were applied to forepaws contralateral to the injured hemisphere. Tactile responses were measured by recording the time to initially contact the paws and the time to remove the adhesive tape. The maximum observation period was 2 minutes.

#### Rotarod Test

Motor coordination and balance dysfunction were tested on an accelerating Rotarod (IITC Life Science). Mice were put on a rotating drum with speed accelerating from 4 to 40 rpm within 5 minutes. Mice were tested 3 times with at least a 10-minute interval. The latency to fall off the rotating rod was recorded. The averages of three trials on the final day of training were recorded as baseline values. The test was repeated at different time points after surgery. Data were expressed as mean values from 3 trials.

#### Cylinder Test

To assess forelimb asymmetry, mice were placed individually inside a transparent cylinder (diameter 9 cm; height 15 cm). A camera was placed above the cylinder to videotape the full vision of the cylinder for 15 minutes. When exploring the cylinder, mice typically exhibit spontaneous rears contacting the cylinder wall with the right forelimb (R), the left forelimb (L), or both forelimbs (B). Data are expressed as asymmetry rate, calculated as (R-L)/(L+R+B) x100%.

#### Hanging Wire Test

The apparatus used was a stainless-steel bar (50 cm long and 2 mm diameter) supported by two vertical supports and elevated 37 cm above a flat surface. Mice were placed in the middle of the bar and were guided to climb onto the supports within 30 seconds. Three trials were performed each day. Mice were scored according to the following criteria: 0, fell off; 1, hung onto the bar with two forepaws; 2, hung onto the bar with an added attempt to climb onto the bar; 3, hung onto the bar with two forepaws and one or both hind paws; 4, hung onto the bar with all four paws and with tail wrapped around the bar; and 5, escaped to one of the supports.

#### Foot Fault Test

Foot-fault is a measurement of forelimb coordination during spontaneous locomotion (15). Mice were placed on a steel grid surface (20 cm x 40 cm with a mesh size of 4 cm^2^) elevated 30 cm above a flat surface. A camera was placed, and the session was videotaped for 1 minute. The videotapes were analyzed by a blinded investigator to count the number of total steps and the number of foot faults made by the impaired limbs (contralateral to lesion). Foot faults were determined when the mouse misplaced its impaired forepaw such that the paw fell through the grid. Data were expressed as a percentage of foot faults among total steps.

### Measurement of Tissue Loss

Animals were euthanized and perfused with saline followed by 4% paraformaldehyde (Sigma-Aldrich) in phosphate-buffered saline (PBS). Brains were cryoprotected in 30% sucrose in PBS. Coronal brain sections (25 μm) were sliced on a freezing microtome (Microm HM 450). Brain sections were subjected to crystal violet staining. Tissue loss was determined using Image J software by an observer blinded to group assignments. The actual tissue loss volumes were calculated as the volume of the contralateral hemisphere minus the non-injury volume of the ipsilateral hemisphere.

### Immunohistochemistry and Image Analysis

Brain sections were blocked with 5% donkey serum in PBS for 1 hour, followed by overnight incubation (4°C) with the primary antibodies. After washing, sections were incubated for 1 hour at 20°C with secondary antibodies conjugated with fluorophores (1:1000, Jackson ImmunoResearch Laboratories, Inc.). Fluorescence images were captured with an Olympus Fluoview FV1000 confocal microscope and FV10-ASW 2.0 software (Olympus America). Primary antibodies used in this study include: Goat anti-IL-33 (R&D System), mouse anti-APC (MilliporeSigma), rabbit anti-GFAP (Dako), mouse anti-NeuN (MilliporeSigma), and rabbit anti-Iba1 (Wako).

### Flow Cytometry

Peripheral blood was collected by cardiac puncture. ACK lysis buffer (Sigma- Aldrich) was used to lyse RBCs. Brains were dissected and the ipsilateral hemispheres were collected. Single-cell suspensions were prepared by using the Neural Tissue Dissociation Kit (Miltenyi Biotec), according to the manufacturer’s instructions. The suspension was passed through a 70-μm cell strainer (Thermo Fisher Scientific) and resuspended in 30% Percoll. Cells, myelin and debris were stratified on a 30–70% Percoll gradient (GE Healthcare BioSciences). Cells at the interface were collected and then washed with FACS buffer (1% penicillin/streptomycin antibiotic, 2% fetal bovine serum, 2 mM EDTA in HBSS) before staining. Cells were first incubated with antibodies to surface antigens for 30 minutes on ice at 4°C in the dark. After two washes, cells were permeabilized and fixed with the Intracellular Staining Kit (Thermo Fisher Scientific), and then stained with fluorophore-labeled antibodies for 30 minutes on ice in the dark. Fluorochrome compensation was performed with single-stained UltraComp eBeads (Thermo Fisher Scientific). Flow cytometry was performed on the BD LSRII flow cytometer (BD Biosciences). Data analyses were performed using FlowJo software. The following antibodies (Thermo Fisher Scientific) were used: anti-CD4 FITC, anti-ST2 APC, anti-CD25 PE, anti-Foxp3 Percp-cy5.5, anti-CD11b APC-cy7, anti-Ly6G FITC, anti-CD3 ef450, anti-CD11c Percp-cy5.5, anti-GITR PE Cy7, anti-CTLA4 BV421, and anti-F4/80 PE.

### Cytokine Enzyme-Linked Immunosorbent Assay

Blood plasma, brain lysates and cell culture media were collected. Protein concentrations were measured with commercial ELISA quantification kits for IL-10 and TGFβ (R&D Systems) according to the manufacturer’s instructions.

### Treg Isolation and Culture

Spleens were harvested from WT or ST2 KO mice 5d after CCI to prepare single cell suspensions as we described (16, 17). CD4^+^CD25^+^ Tregs were isolated using a mouse Treg isolation kit (Miltenyi Biotec) according to the manufacturer’s instructions. The isolation was performed in a two-step procedure with a negative selection on CD4^+^ cells and a positive selection on CD25^+^ cells. Isolated Tregs were stimulated with soluble anti-CD3 (4 µg/ml), anti-CD28 (5 µg/ml) and IL2 (100 ng/ml) for 2d in Treg culture media (RPMI1640 containing 2mM L-Glutamine, 10%FBS, 1% penicillin/streptomycin, 1mM pyruvate sodium, and 55 μm β-mercaptoethanol) and then treated with IL-33 (R&D system, 50 ng/mL) or PBS for 24h.

### BrdU Proliferation

T effector cells (Teff) were plated at 2 × 10^5^ per well in a U bottom 96-well plate in the presence of anti-CD3 and anti-CD28 to stimulate their proliferation. IL-33 treated or PBS-treated Tregs were added at a ratio of 1:1, 1:2, 1:4, 1:8, and 1:16 to the number of Teff. Cells were incubated for two days. Suppression of Teff proliferation was determined using the BrdU cell proliferation ELISA kit (Roche) according to the manufacturer’s instructions.

### Statistical Analyses

All statistical analyses were performed using GraphPad Prism software (version 9.0.0, La Jolla, CA). The Student’s t-test was used for comparison of two groups for continuous variables with normal distributions. The differences in means among multiple groups were analyzed using one-way analysis of variance (ANOVA). Differences in means across groups with repeated measurements over time were analyzed using the two-way repeated measures ANOVA. When the ANOVA showed significant differences, pairwise comparisons between means were tested by *post hoc* Bonferroni multiple-comparison tests. Results are presented as mean ± SEM. In all analyses, p<0.05 was considered statistically significant.

## Results

### TBI Results in Increased Expression of IL-33 and ST2

**Figure 1:**
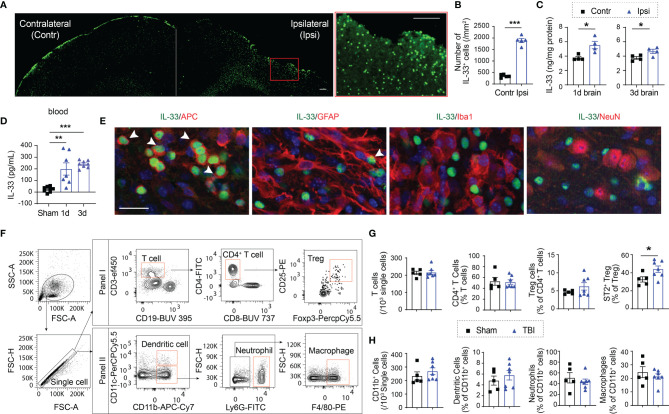
TBI results in increased expression of IL-33 and ST2. **(A)** Representative images of IL-33 (green) staining in a brain slice collected 5d after CCI. More IL-33^+^ cells were observed in the lesioned side (ipsilateral) of the brain. The region enclosed by the red box in the left image was enlarged in the right image. Scale bar, 150 μm. **(B)** Quantification of IL-33^+^ cells in the ipsilateral lesioned area and corresponding contralateral brain area 5d after CCI. **(C, D)** IL-33 protein levels were measured in the brain lysates **(C)** and blood **(D)** collected 1 and 3 days after CCI. **(E)** Double staining of IL-33 (green) in APC^+^ oligodendrocytes (red), GFAP^+^ astrocytes (red), Iba1^+^ microglia/macrophages (red) and NeuN^+^ neurons (red) in ipsilateral brains 5 days after CCI. Scale bar, 40 μm. Nuclei were stained blue with 4′,6-diamidino-2-phenylindole (DAPI). All images are representative of four animals. **(F)** Representative gating strategy for T lymphocytes, monocytes, neutrophils, and dendritic cells in the blood 5d after CCI. **(G)** Quantification of total CD3^+^ T cells, CD4^+^ T cells, CD4^+^CD25^+^Foxp3^+^ Tregs, and ST2^+^ Tregs in blood 5d after CCI or sham operation. **(H)** Quantification of total CD11b^+^ cells, CD11c^+^CD11b^+^ dendritic cells, CD11b^+^Ly6G^+^ neutrophils, and CD11b^+^F4/80^+^ macrophages in blood 5d after CCI or sham operation. *p < 0.05, **p < 0.001, ***p < 0.001, Student’s t test **(B, C, G, H)** or one-way ANOVA and *post hoc* Bonferroni **(D)**.

We evaluated IL-33 expression in the brain and the blood after CCI. Immunostaining showed that the number of IL-33^+^ cells increased significantly 5d after CCI (**Figures 1A, B**). ELISA results confirmed elevated IL-33 levels in the ipsilateral brain (**Figure 1C**) and in the plasma (**Figure 1D**) 1d and 3d after CCI. Co-labeling with cell specific markers demonstrated IL-33 protein expression mainly in APC^+^ oligodendrocytes in the lesioned area (**Figure 1E**). Some IL-33^+^GFAP^+^ astrocytes were noted (**Figure 1E**). There is no IL-33 staining in Iba1^+^ microglia/macrophages or NeuN^+^ neurons (**Figure 1E**).

We then assessed changes in immune cell composition in blood after TBI (**Figure 1F**). We did not see significant changes in the numbers of CD3^+^ total T lymphocytes (**Figure 1G**), CD19^+^ B lymphocytes (not shown), or CD11b^+^ myeloid cells (**Figure 1H**). The percentages of CD11b^+^CD11c^+^ dendritic cells, CD11b^+^Ly6G^+^ neutrophils or CD11b^+^F4/80^+^ macrophages remained unchanged among total CD11b^+^ myeloid cells 5d after TBI (**Figure 1H**). Meanwhile, the percentages of CD4^+^ T cells and CD4^+^CD25^+^Foxp3^+^ Tregs showed no change in blood (**Figure 1G**). Interestingly, the percentage of ST2^+^ Tregs significantly increased in the blood 5d after TBI (**Figure 1G**). There is no significant change in ST2 expression in total CD4^+^ T cells (not shown).

**Figure 2:**
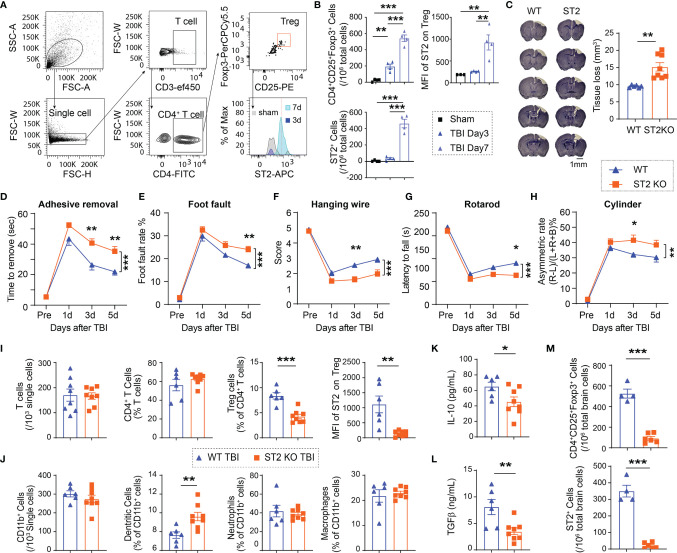
ST2 knockout (KO) reduces Treg frequencies, enlarges brain lesion size and exacerbates functional deficits after TBI. **(A)** Representative gating strategy for CD4^+^ T cells, CD4^+^CD25^+^Foxp3^+^ Tregs, and ST2^+^ Tregs in the WT brain after CCI. **(B)** Quantification of CD4^+^CD25^+^Foxp3^+^ Tregs, ST2^+^ Tregs, and median fluorescence intensity (MFI) of ST2 in Tregs in the sham WT brain and WT brains collected 3d and 7d after CCI. n = 4/group. (C-M) Male C57/BL6 WT and ST2 KO mice were subjected to CCI. n = 6-8/group. **(C)** Representative images of crystal violet-stained brain sections collected 5d post-injury in WT and ST2 KO mice. Brain tissue loss was quantified. **(D–H)** Sensorimotor functions after CCI were assessed in WT and ST2 KO mice. **(D)** Adhesive removal test. The latency to remove the tape from the impaired forepaw was recorded. **(E)** Foot fault test was quantified as foot fault rate, which is the ratio of the total number of foot faults for the left forelimb to the total movement number of the left forelimb. **(F)** Hanging wire test. **(G)** Rotarod test. The latency to fall off the rotating rod was recorded. **(H)** Cylinder test. The asymmetric rate was calculated as described in Methods. **(I)** Quantification of total CD3^+^ T cells, CD4^+^ T cells, CD4^+^CD25^+^Foxp3^+^ Tregs, and MFI of ST2 in Tregs in the blood 5d after CCI in WT and ST2 KO mice. **(J)** Quantification of total CD11b^+^ cells, CD11c^+^CD11b^+^ dendritic cells, CD11b^+^Ly6G^+^ neutrophils, and CD11b^+^F4/80^+^ macrophages in the blood 5d after CCI in WT and ST2 KO mice. **(K, L)** Plasma IL-10 **(K)** and TGFβ (L) levels were measured by ELISA 5d after CCI in WT and ST2 KO mice. (M) Quantification of CD4^+^CD25^+^Foxp3^+^ Tregs and ST2^+^ Tregs in the brain 5d after CCI in WT and ST2 KO mice. *p < 0.05, **p < 0.01, ***p < 0.001, Student’s t test **(C, I, J, K, L, M)**, one-way ANOVA **(B)** or two-way repeated measures ANOVA **(D–H)** and *post hoc* Bonferroni.

We then assessed the infiltration of Tregs in the TBI brain. Infiltrating Tregs were observed in the brain 3d after CCI, and further increased 7d after CCI. The number of ST2^+^ Tregs significantly increased in the brain 7d after CCI (**Figures 2A, B**). ST2 expression in infiltrating Tregs increased since 7d after CCI (**Figures 2A, B**).

Taken together, these results demonstrate that IL-33 is released in the brain and in the circulation after TBI, which is accompanied by ST2 upregulation in circulating and brain infiltrating Tregs.

### ST2 Deficiency Enlarges Brain Lesion Size, Exacerbates Functional Deficits, and Reduces Treg Frequencies After CCI

We then used ST2 knockout mice to investigate the function of IL-33/ST2 axis after TBI. ST2 KO mice exhibited enlarged brain lesion size 5d after CCI (**Figure 2C**). ST2 deficiency also resulted in deteriorated sensorimotor deficits, as revealed by the increased time to remove an adhesive tape in the adhesive removal test (**Figure 2D**), increased error rate in the foot fault test (**Figure 2E**), lower score in hanging wire test (**Figure 2F**), reduced latency to fall off a rotating bar in the rotarod test (**Figure 2G**), and higher asymmetric rate in the cylinder test (**Figure 2H**). Flow cytometry results showed no significant differences in the numbers of circulating Tregs, as well as total T lymphocytes, CD4^+^ T lymphocytes, CD11b^+^ myeloid cells, or CD11b^+^Ly6G^+^ neutrophils between ST2 KO sham mice and WT sham mice (**Figure S1**). However, significantly reduced ST2 expression in circulating Tregs was observed, which was accompanied by a reduced number of Tregs in the blood in ST2 KO mice *vs.* wild-type (WT) mice 5d after CCI (**Figure 2I**). The percentages of CD11b^+^CD11c^+^ dendritic cells increased significantly in ST2 KO mice after CCI (**Figure 2J**). The numbers of other immune cells were similar between the two genotypes (**Figure 2I, J**). Consistent with the drop in Treg numbers, the expression of IL-10 (**Figure 2K**) and TGFβ (**Figure 2L**), two anti-inflammatory cytokines released by Tregs, was significantly reduced in ST2 KO mice compared to WT mice 5d after CCI. ST2 deficiency also reduced the numbers of brain infiltrating Tregs and ST2^+^ infiltrating Tregs 5d after CCI (**Figure 2M**).

### IL-33 Treatment Reduces Brain Lesion and Functional Deficits, and Adjusts Peripheral Treg Responses After CCI

**Figure 3:**
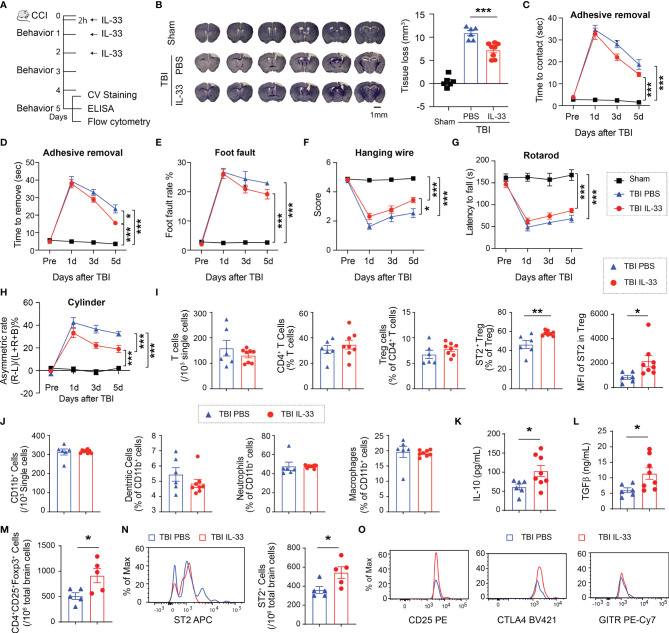
IL-33 treatment reduces brain lesion and functional deficits after TBI and adjusts peripheral Treg responses. **(A)** Experimental design. C57/BL6 mice were treated with IL-33 (2 μg/30 g body weight) or the same volume of PBS vehicle intranasally 2h after CCI and repeated daily for 2 more days. n = 6-8/group. **(B)** Tissue loss was evaluated in coronal brain sections stained by crystal violet 5d after TBI. Representative images show six crystal violet-stained brain sections, spanning from 1.10 mm anterior to bregma to 1.94 mm posterior to bregma with the same interval. **(C–H)** Sensorimotor function was evaluated by the adhesive removal test **(C, D)**, foot fault test **(E)**, hanging wire test **(F)**, rotarod test **(G)** and the cylinder test **(H)**. **(I)** Flow cytometry quantification of total CD3^+^ T cells, CD4^+^ T cells, CD4^+^CD25^+^Foxp3^+^ Tregs, ST2^+^ Tregs, and MFI of ST2 in Tregs in blood 5d after CCI. **(J)** Quantification of total CD11b^+^ cells, CD11c^+^CD11b^+^ dendritic cells, CD11b^+^Ly6G^+^ neutrophils, and CD11b^+^F4/80^+^ macrophages in the blood 5d after TBI. **(K–L)** Plasma IL-10 **(K)** and TGFβ **(L)** levels were measured by ELISA 5d after CCI. **(M–N)** Quantification of CD4^+^CD25^+^Foxp3^+^ Tregs **(M)** and ST2^+^ Tregs in the brain 5d after CCI. **(O)** Expression levels of CD25, CTLA4 and GITR in Tregs in the brain 5d after CCI. *p < 0.05, **p < 0.01, ***p < 0.001. Student’s t test **(I–N)**, one-way **(B)** or two-way repeated measures ANOVA **(C–H)** and *post hoc* Bonferroni.

We then treated mice with IL-33 (2 μg/30 g body weight) intranasally starting 2h after CCI and repeated for 2 consecutive days (**Figure 3A**). In contrast to ST2 deficiency, the brain lesion size significantly reduced in IL-33-treated mice compared to PBS-treated mice (**Figure 3B**). The supplementation of IL-33 improved sensorimotor functions in some behavioral tests including the adhesive removal test (**Figures 3C, D**), the hanging wire test (**Figure 3F**), and the cylinder test (**Figure 3H**) up to 5d after CCI. IL-33 did not significantly improve the performance in foot fault test (**Figure 3E**) or rotarod test (**Figure 3G**). IL-33 treatment did not change the number of circulating Tregs 5d after TBI (**Figure 3I**) but increased the percentage of ST2^+^ Tregs and the expression level of ST2 on Tregs in the blood (**Figure 3I**). The percentages of CD11b^+^CD11c^+^ dendritic cells, CD11b^+^Ly6G^+^ neutrophils and CD11b^+^F4/80^+^ macrophages remained the same among total CD11b^+^ myeloid cells in IL-33 and PBS-treated mice 5d after TBI (**Figure 3J**). IL-33 increased the levels of IL-10 (**Figure 3K**) and TGFβ (**Figure 3L**) in the blood 5d after TBI. IL-33 treatment increased the number of brain infiltrating Tregs (**Figure 3M**) and ST2^+^ infiltrating Tregs (**Figure 3N**). The expression levels of CD25, CTLA4 and GITR on infiltrating Tregs did not show significant differences 5d after TBI (**Figure 3O**).

These data suggest that IL-33 reduces brain lesion and sensorimotor deficits after CCI and enhances anti-inflammatory response, possibly by upregulating ST2 expression on Tregs.

### Treg Is Essential for Neuroprotective Effect of the IL-33/ST2 Signaling

**Figure 4:**
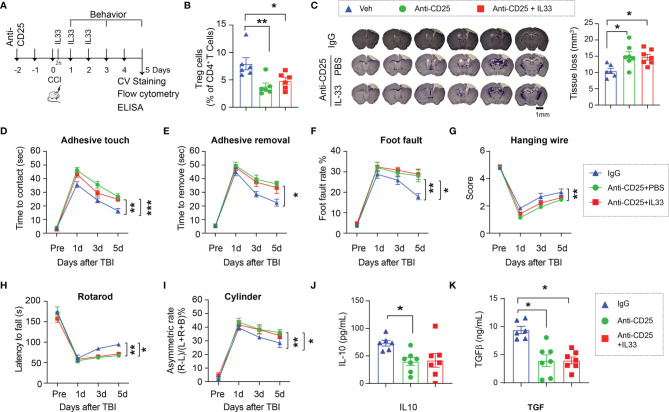
Treg is essential for the neuroprotective effect of the IL-33/ST2 axis. **(A)** Experimental design. C57/BL6 mice were treated with isotype IgG Veh (300 µg) or anti-CD25 mAb (300 µg) 2d prior to CCI. Some anti-CD25 mAb-treated mice received IL-33 (2 μg/30 g body weight) intranasally 2h after CCI and repeated daily for two more days. n = 6-7/group. **(B)** Flow cytometry confirms the reduction of Tregs in blood of CD25 Ab treated mice 5d after CCI. **(C)** Left: representative brain sections of crystal violet staining show the tissue loss 5d post TBI. Right: Quantification of total volume of tissue loss. **(D–I)** Sensorimotor function was evaluated by the adhesive removal test **(D, E)**, foot fault test **(F)**, hanging wire test **(G)**, rotarod test **(H)**, and the cylinder test **(I)**. **(J, K)** Plasma IL-10 **(J)** and TGFβ **(K)** levels were measured by ELISA 5d after CCI. *p < 0.05, **p < 0.01, ***p < 0.001. One-way **(B, C, J, K)** or two-way repeated measures ANOVA **(D–I)** and *post hoc* Bonferroni.

Since ST2 is widely expressed in multiple types of immune cells, we next confirmed whether Tregs are essential for the protective effect of the IL-33/ST2 signaling early after TBI. Tregs were depleted by anti-CD25 injection 48 hours prior to TBI (**Figure 4A**). As expected, anti-CD25 injected mice maintained lower numbers of Tregs in the circulation regardless of IL-33 treatment 5d after CCI (**Figure 4B**). Treg depletion enlarged brain lesion size and exacerbated sensorimotor deficits, and IL-33 injection to Treg-depleted mice lost its protective effect (**Figures 4C–I**). The expression of IL-10 (**Figure 4J**) and TGFβ (**Figure 4K**) remained in low levels in Treg depleted mice with or without IL-33.

### IL-33 Enhances the Production of Anti-Inflammatory Cytokines From Tregs and Boosts Their Suppressive Effects on T Effector Cells

**Figure 5:**
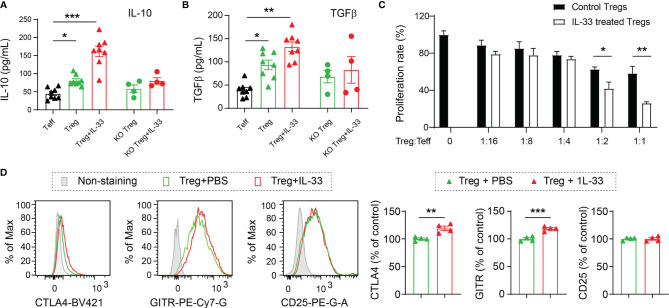
IL-33 enhances the production of anti-inflammatory cytokines from Tregs and boosts their suppressive effects on T effector cells. Tregs and Teff were prepared from the spleens collected from the WT or ST2 KO mice 5d after CCI. Tregs were cultured with anti-CD3 and anti-CD28 for 2 days followed by IL-33 (50 ng/ml) or PBS treatment for 24h. **(A, B)** IL-10 **(A)** and TGFβ **(B)** levels in conditioned media were measured by ELISA. One-way ANOVA and *post hoc* Sidak. **(C)** The effect of Tregs on Teff proliferation was measured by BrdU incorporation. Vehicle or IL-33 treated Tregs were added at a ratio of 1:1, 1:2, 1:4, 1:8, and 1:16, to the number of Teff. Cells were incubated for 2d. Suppression of Teff proliferation was determined using a BrdU cell proliferation kit. n = 8 per condition. **(D)** Flow cytometry analysis of CTLA4, GITR and CD25. n = 4. Two-way ANOVA and *post hoc* Bonferroni **(A–C)** and Student’s t test **(D)**. *p < 0.05, **p < 0.01, ***p < 0.001.

To elucidate the direct effect of IL-33 on Tregs, we isolated Tregs from the spleens of WT and ST2 KO mice at 5d after CCI and maintained in culture media with anti-CD3 and anti-CD28 for 2 days. Activated Tregs were then cultured for 24h with or without IL-33 treatment. T effector cells (Teff) were used as control. As shown in **Figures 5A, B**, there were higher levels of IL-10 and TGFβ in the conditioned media collected from Tregs compared to Teff cells. IL-33 treatment further increased the expression of these two cytokines. IL-33 failed to induce IL-10 or TGFβ production in ST2 KO Tregs (**Figures 5A, B**).

The capacity of Tregs to inhibit the proliferation of Teff cells was measured by the BrdU cell proliferation kit (**Figure 5C**). IL-33-treated WT Tregs demonstrated enhanced ability to inhibit the proliferation of Teff cells, as shown by significantly reduced BrdU incorporation (**Figure 5C**).

We also measured the expression of effector molecules CTLA4, GITR and CD25. IL-33 treatment enhanced the expression of CTLA4 and GITR in cultured Tregs but showed no effect on CD25 expression (**Figure 5D**).

## Discussion

IL-33 is known as an alarming protein that is released from damaged cells and triggers subsequent immune responses. The elevation of IL-33 in lesioned brains has been observed in different CNS injuries including TBI (18), stroke (6), spinal cord injury (19), and multiple sclerosis (20). Samples from human TBI microdialysate and brain sections show increases in IL-33 levels early after TBI (18). Consistent with this study, we found that IL-33 levels increased in the brain 1 and 3 days after CCI. The main cellular sources of IL-33 in the injured CNS are diverse and seem to be different according to specific injuries. Oligodendrocytes and astrocytes are the main sources of IL-33 in stroke and TBI brains (6, 18). IL-33 is mainly localized in astrocytes after spinal cord injury (19), while being widely expressed in microglia, neurons, oligodendrocytes and astrocytes in multiple sclerosis (20). In our study, there is a predominant expression of IL-33 in oligodendrocytes at the site of injury after TBI. This is consistent with the relatively high basal level of IL-33 expression in oligodendrocytes in a normal brain (21). Interestingly, double staining of IL-33 with oligo2, a marker for whole lineage of oligodendrocytes, shows less percentages of colocalization than that in APC^+^ mature oligodendrocytes (not shown), suggesting that this alarmin protein upsurges preferentially in mature oligodendrocytes after TBI.

Previous studies about the IL-33/ST2 signaling in brain injuries mainly focus on its effects within the CNS. Microglia/macrophages have been identified as the main cellular targets of IL-33 in stroke models (6, 7). In addition, approximately 50% of the brain infiltrating Tregs express ST2 and expand in response to IL-33 after brain ischemia (9). Consistent with these studies in stroke models, we found reduced number of brain infiltrating Tregs in ST2 deficient mice and increased number of brain infiltrating Tregs in IL-33 treated mice after TBI. Therefore, IL-33/ST2 signaling is important for Treg recruitment and/or amplification in the brain in different CNS injuries.

In addition to its central functions, a high level of IL-33 is noted in blood early after TBI in numerous clinical reports (12, 13, 22). The ascending serum IL-33 levels correlate with clinical severity and could be used as a marker for risk assessments and prognosis in TBI patients. The function of circulating IL-33 after TBI is unknown. In this study, we found an increase of IL-33 in the blood as early as 1d after CCI, which remained high for at least 3 days. We further found that the ST2 expression on circulating Tregs increased after TBI and its expression on brain infiltrating Tregs escalated with time. Our experiments using IL-33 supplementation and ST2 KO mice revealed the impact of this signaling axis on Treg responses after TBI. IL-33 increases ST2 expression on circulating and infiltrating Tregs; and ST2 expression maintains Treg numbers in the blood and in the brain after TBI. IL-33 also enhanced the expression of Treg activation markers CTLA4 and GITR in cultured Tregs, but not in brain infiltrating Tregs after TBI. It seems that some effects of IL-33 on Tregs are subdued by other stimulations in a complicated *in vivo* environment. It has been reported that ST2 or IL-33 deficiency has no effect on the frequencies and total numbers of Tregs in peripheral lymph organs in normal mice (23). In agreement of this report, we found that the number of circulating Tregs in ST2 KO sham mice did not change compared to WT sham mice. Therefore, the activation of IL-33/ST2 signaling axis might be a mechanism to induce protective Treg responses under pathological conditions including TBI but has little impact on Treg generation or differentiation under normal conditions.

Clinical studies have shown that the number of circulating Tregs increases transiently on the first day after TBI, followed by a decrease on the 4^th^ day and a rebound 7-14 days after TBI (24). Importantly, the number of circulating Tregs correlates with clinical recovery. TBI patients with circulating Tregs more than 4.91% of total CD4^+^ cell numbers in the first 14 days after TBI exhibit better neural recovery compared to those with lower numbers of Tregs (24). Evidence in the pre-clinical models supports beneficial effects of Tregs in acute tissue protection after TBI (25). Consistent with previous study, we found that depletion of Tregs enlarged brain lesion and profoundly exacerbated functional outcomes 5d after TBI. Indeed, we showed that IL-33 failed to provide protection to Treg-depleted mice early after TBI. In line with this notion, Tregs have also been shown to be critical for the beneficial effects of IL-33 in the ischemic brain (9, 11).

The IL-33/ST2 pathway may regulate Treg responses through different mechanisms. For example, ST2 signaling in Treg cells induces the expression of Ebi3, a component of the anti-inflammatory cytokine IL-35 and enhances Treg-mediated suppression of γδ T cells in the lungs (26). Another study showed that the IL-33/ST2 signaling axis is required to promote the production of type 2 cytokine, including IL-13 and IL-5, by Tregs in adipose tissue (27). Here, we found that IL-33/ST2 signaling is important for the production of IL-10 and TGFβ, two anti-inflammatory cytokines that have been shown to be protective in TBI models (28, 29) from Tregs. The lack of IL-33/ST2 axis impaired the production of Treg-derived IL-10 and TGFβ and led to exacerbated brain lesions and neurological deficits. Thus, the IL-33/ST2 engagement not only boosts the number of Tregs, but also shapes the secretory profile of Tregs in different tissues or in response to different stimulations.

Although anti-CD25 antibody is a commonly used approach for Treg depletion, (30, 31) we are aware that one shot of anti-CD25 cannot completely deplete all Tregs (32). Significant reduction in the number of Tregs is observed at 5d after TBI, which is the main time points for our endpoint evaluation. Therefore, it is valid to use anti-CD25 as a loss of function model to assess the effect of Tregs in acute TBI.

Since ST2 is widely expressed in many types of CNS cells and peripheral immune cells, it is conceivable that IL-33 released after TBI may target other types of cells to exert its protective functions. For example, it is reported that IL-33 can recruit microglia/macrophages to the site of injury after TBI (18). Our previous studies in stroke model show an effect of IL-33/ST2 signaling in promoting an anti-inflammatory microglia phenotype (6). In our study, we also noticed an increase in the number of dendritic cells in the blood of ST2 KO mice. It might be a direct effect of IL-33 on dendritic cells as ST2 is expressed on this cell type, or an indirect compensatory response to the changes in Tregs (33). In addition. ST2 is shown to be expressed in oligodendrocytes, astrocytes and neurons (34). Therefore, the mechanisms of IL-33/ST2-afforded protection against TBI might be multifaceted. Further studies are warranted to elucidate a full cell-cell interaction network initiated by the IL-33/ST2 signaling. Our studies using Treg depleted mice and *in vitro* Treg cultures unequivocally confirm Tregs as an important element in this network.

In summary, this study demonstrates that the activation of IL-33/ST2 signaling protects against brain injury and reduces neurological deficits after TBI through modulating Treg responses. IL-33 treatment or other approaches that boost Treg number or function may serve as therapeutic strategies to restrict acute brain injury after TBI and thus improve long-term outcomes.

## Data Availability Statement

The raw data supporting the conclusions of this article will be made available by the authors, without undue reservation.

## Ethics Statement

The animal study was reviewed and approved by the University of Pittsburgh Institutional Animal Care and Use Committee. Written informed consent was obtained from the owners for the participation of their animals in this study.

## Author Contributions

DX, WM, CY, SL, CW, and AJ performed the *in vivo* experiments and data analyses. DX, FX, and CY performed the *in vitro* culture experiments and data analysis. DX and CW maintained animal breeding. DX, WM, CW, AJ, and XH wrote the manuscript. XH designed and supervised the study. All authors contributed to the article and approved the submitted version.

## Funding

DX is a visiting scholar from The Third Xiangya Hospital, Central South University, Changsha, China. All work was performed at the University of Pittsburgh and was supported by funding from the University of Pittsburgh. XH is supported by VA Merit Review grants (I01BX003651 and I01BX005589).

## Conflict of Interest

The authors declare that the research was conducted in the absence of any commercial or financial relationships that could be construed as a potential conflict of interest.

## Publisher’s Note

All claims expressed in this article are solely those of the authors and do not necessarily represent those of their affiliated organizations, or those of the publisher, the editors and the reviewers. Any product that may be evaluated in this article, or claim that may be made by its manufacturer, is not guaranteed or endorsed by the publisher.
