# Supplementary material for: IL-33/ST2 Axis Protects Against Traumatic Brain Injury Through Enhancing the Function of Regulatory T Cells
Source: Front Immunol. 2022 Mar 30;13:860772. doi: 10.3389/fimmu.2022.860772 (PMC9006950; doi:10.3389/fimmu.2022.860772)
Supplement: Supplementary file 1 [file DataSheet_1.pdf]

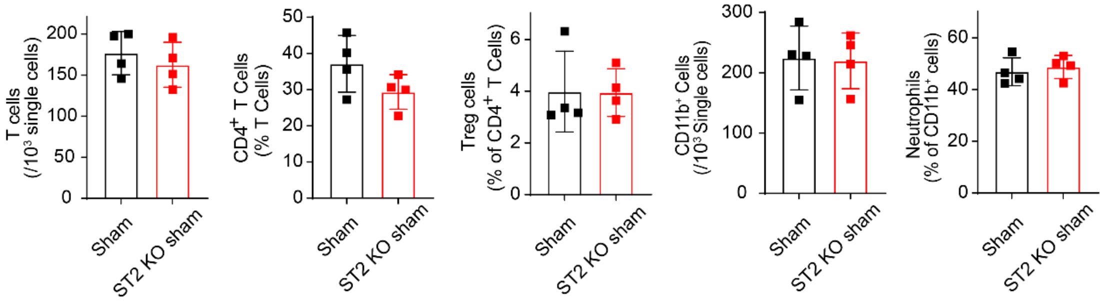

Figure S1. Immune cell assessment in sham ST2 KO and sham WT mice. Quantification of total $CD3^+$ T cells, $CD4^+$ T cells, $CD4^+CD25^+Foxp3^+$ Tregs, total $CD11b^+$ cells, and $CD11b^+Ly6G^+$ neutrophils in blood. n=4.
